# A Novel Method for the Locomotion Control of a Rat Robot via the Electrical Stimulation of the Ventral Tegmental Area and Nigrostriatal Pathway

**DOI:** 10.3390/brainsci15040348

**Published:** 2025-03-27

**Authors:** Bo Li, Honghao Liu, Guanghui Li, Yiran Lang, Rongyu Tang, Fengbao Yang

**Affiliations:** 1 School of Information and Communication Engineering, North University of China, Taiyuan 030051, China; yangfb@nuc.edu.cn; 2 School of Mechatronical Engineering, Beijing Institute of Technology, Beijing 100081, China; 3120205085@bit.edu.cn; 3 Department of Neuroscience, Faculty of Health and Medical Sciences, University of Copenhagen, DK-2200 Copenhagen, Denmark; guanghui.li@sund.ku.dk; 4 School of Medical Technology, Beijing Institute of Technology, Beijing 100081, China; yiran.lang@bit.edu.cn; 5 Institute of Semiconductors, Chinese Academy of Sciences, Beijing 100083, China; tangrongyu@semi.ac.cn

**Keywords:** rat robot, electrical stimulation, ventral tegmental area, nigrostriatal pathway, inclined movement, turning behavior

## Abstract

Background: A rat robot can be constructed by electrically stimulating specific brain regions to control rat locomotion and behavior. The rat robot makes full use of the rat’s motor function and energy supply and has significant advantages in motor flexibility, environmental adaptability, and covertness. It can be widely used in disaster search and rescue, terrain survey, anti-terrorism, and explosion-proof tasks. However, the motor control of existing rat robots mainly relies on the virtual whisker touch produced by the electrical stimulation of the barrel area of the somatosensory cortex and the virtual reward generated by the electrical stimulation of the medial forebrain bundle. The methods requires substantial experimental training to encourage the animals to match the virtual sensation with the motor behavior. However, the conditioned reflexes acquired by the animals will gradually disappear after a period of time at the end of the experiments, which will lead to a decrease in the stability of the motor control system. Methods: In this study, we developed a new method to gain control of inclined movement in rats by the electrical stimulation of the ventral tegmental area (VTA) of the midbrain and motor control of steering in rats by the electrical stimulation of nigrostriatal (NS) pathway. Results: The results showed that the electrical stimulation of the rat VTA could induce stable inclined movement in rats and that the neuromodulatory effect significantly correlated with the electrical stimulation parameters. In addition, the electrical stimulation of the NS pathway was able to directly and stably induce the steering movements of the head and trunk to the contralateral side of the stimulated side of the rat. Conclusions: These findings are of great importance for the motor control of rat robots, especially in the field environment with many slopes. In addition, the rat robot constructed based on this method does not need pre-training while ensuring reliability, which greatly improves the preparation efficiency and has certain practical application value.

## 1. Introduction

Animal robots use a brain–computer interface as a medium to neuromodulate the sensory-motor system of animals to gain control of their movement, and with the help of the animals’ own motor function and energy supply system, the animals will be prompted to perform all kinds of tasks that are not easy for humans to perform due to the human’s intentions. Compared to traditional robots, the animal body itself serves as a carrier and power source, obviating the need for traditional robot research and the development of structural dynamics design and weight, energy supply optimization, and other difficult problems, thus greatly reducing the difficulty and cost of system development. Moreover, animal robots have natural advantages in mobility, flexibility, environmental adaptability, camouflage, and stealth, which can be widely used in hazardous environment search and rescue operations, anti-terrorism reconnaissance, and confined space operations. Therefore, many animals have been used to build robots, including cockroaches [1], bees [2], beetles [3], sharks [4], geckos [5], pigeons [6], etc. Among these animal robots, the study of insect robots is currently a research hotspot. The implementation of insect flight-inspired control strategies in robots solves important technical bottlenecks, such as miniaturization, flight stability, and the power supply of bionic vehicles, and has led to better concealment, which has great potential for application in military reconnaissance and other fields. However, the size of the insect itself determines its limited carrying capacity, and it is difficult to equip other surveillance equipment after carrying the control equipment, which severely limits its applicability in practical scenarios.

Comparatively speaking, as a rodent mammal, the rat has characteristics of strong locomotion, high flexibility, high stealth, and strong environmental adaptability and can move back and forth in a small space and is good at night activities, so it can be widely used in confined space detection, terrain surveying, fire rescue, and other scenarios. However, its more complex motion control system also presents more challenges for effective motion control. Based on the virtual tactile evocation principle, steering movements were induced by electrically stimulating the barrel field of the primary somatosensory cortex (S1BF) on both sides of the rat brain to generate virtual haptic sensations of touching an obstacle, while the medial forebrain bundle (MFB) was electrically stimulated to provide reward reinforcement to gain control of forward movements [7,8]. Based on the motor modulation principle of virtual punishment, fear responses were induced by electrically stimulating the ventral posterolateral thalamic nucleus and the amygdala nucleus on both sides of the rat brain to gain directional control [9]. The stop behavior was achieved by electrically stimulating the dorsolateral periaqueductal gray region (dlPAG) of the rat’s midbrain to induce defensive behavior, and the action–stop state switching and steering movements were achieved by combining the electrical stimulation of the dlPAG with the electrical stimulation of the MFB and S1BF brain regions [10]. Subsequent studies have proposed electrical stimulation of the ventral posteromedial thalamic nucleus to gain rat locomotor control, which have further improved the control efficiency of rat locomotion [11,12]. The researchers then introduced deep learning models into the navigation control of the rat robot, greatly reducing the reliance on manually given commands [13,14,15]. In contrast to electrical stimulation, the stimulation of channelrhodopsin-2 (ChR2) channel protein-transfected dlPAG neurons with light of a specific intensity and frequency induced stop escape behavior in rats [16,17,18].

However, the existing turning motor control of the rat robot mainly relies on the virtual whisker touch sensation generated by the electrical stimulation of the S1BF and the virtual reward generated by the electrical stimulation of the MFB. The implementation of such motor control requires substantial experimental training to induce the animals to match the virtual sensation with their motor behaviors. Moreover, such conditioned reflexes gradually disappear after a period of time at the end of the experiment, leading to a decrease in the stability of motor control. The elicitation of forward locomotor behavior makes use of the rat’s own experience and instinct more than the direct regulation of locomotor behavior, which is prone to inefficiency. Therefore, the main purpose of this paper was to explore new brain regions for motor function control, as well as more direct and reward-independent stimulation modes, in order to gain motor control in brain-controlling biological rats without the need for advanced training.

Previous research on rat robotics has shown that the electrical stimulation of the MFB induces a sense of pleasure, which in turn induces an increase in locomotor activity, resulting in “forward” movements in rats [7]. However, the MFB is only an intermediate pathway of the rat’s reward system, which is essentially a bundle of nerve fibers composed of axons from projection neurons in upstream brain regions, not a complete cerebral nucleus. Therefore, the core control region for the forward commands of a rat robot should be the upstream projection brain region of the MFB, and the ventral tegmental area (VTA) is the upstream projection brain region of the MFB. Additionally, the VTA is thought to be the initiating brain region of the reward system, sending dopamine (DA) to other brain regions and modulating motor activity in animals [19,20]. Thus, increasing motor activity by modulating neural activity in rewarding brain regions may be an effective means of modulating forward motor behavior. The nigrostriatal (NS) pathway is a DA pathway in the brain which is involved in motor control as part of the basal ganglia motor circuit [21,22,23,24]. Previous studies have shown that the electrical stimulation of the NS pathway elicits transient reversal or turning movements in rats and cats [25,26,27], but its modulatory effect as an inducer of turning movements in rats remains to be fully elucidated. We hypothesized that the electrical stimulation of the NS pathway may be a more direct reward-independent mechanism of controlling motor control in rats. Therefore, in this study, we investigated the modulatory effects of the electrical stimulation of the VTA to control the inclined movement and electrical stimulation of the NS pathway to control turning movement in rats, aiming to develop a reward-free method for controlling robotic locomotion in rats.

## 2. Materials and Methods

### 2.1. Experimental Animals

Six 8-week-old male Sprague Dawley (SD) rats (weight: 300 ± 10 g) were used in this study. Rats were housed in a plexiglass cage (36 cm long, 20 cm wide, and 21 cm deep) with sawdust bedding and a 12:12 h light–dark cycle with lights on at 6:00 AM. The rats had free access to food and water throughout the experiment. All animals were housed individually and were given ad libitum food and supplemented with dietary gel to facilitate recovery and minimize tissue damage for 3 days after the surgery. Additional soft bedding and nesting material were provided during recovery to mitigate stress. At the end of all experiments, rats were sacrificed by intraperitoneal injections of Phenobarbitone. The experiment was conducted at the Beijing Institute of Technology (Beijing, China) and was approved by the Institutional Animal Care and Use Committee of Beijing Institute of Technology. These animal experiments complied with the ARRIVE guidelines and were performed in accordance with the U.K. Animals (Scientific Procedures) Act 1986 and associated guidelines.

### 2.2. Surgical Procedures

**Figure 1:**
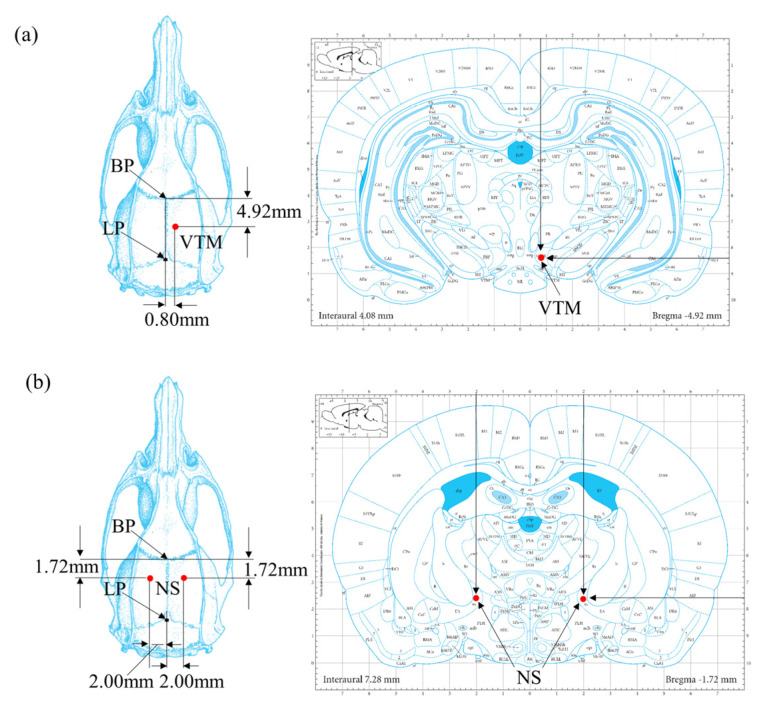
The coordinates of the electrode implantation points. (**a**) The implantation points in the VTA brain region. (**b**) The implantation points in the right and left NS brain regions.

Electrode implantation was performed in a sterile environment under gas anesthesia using 2 to 3% isoflurane gas. After shaving each rat’s head, the scalp was sterilized with iodine and alcohol, and the scalp was removed to expose the skull. The coordinates of the electrode implantation points were then determined according to the standard rat brain stereotaxic atlas, including the implantation points in the VTA brain region (AP = −4.92 mm, ML = −0.80 mm, DV = −8.50 mm) (Figure 1a) and the implantation points in the right and left NS brain regions (AP = −1.72 mm, ML = ±2.00 mm, DV = −7.60 mm) (Figure 1b). All coordinates were based on stereotaxic coordinates from the bregma point.

**Figure 2:**
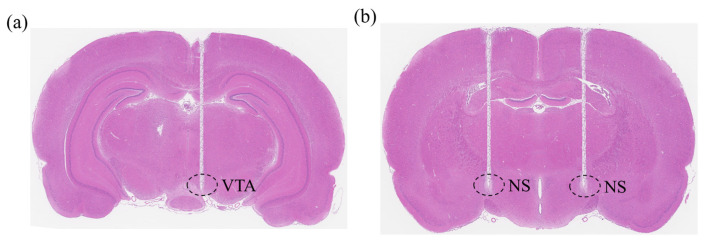
The histological analysis of the rat brain tissue. (**a**) Histological evaluation of the implanted electrodes in the VTA. (**b**) Histological evaluation of the implanted electrodes in the NS pathway.

The electrodes used in the experiments were concentric bipolar stimulation electrodes purchased from Kedou Brain Computer Technology Co., Ltd. (Suzhou, China). The electrode material was a nickel–titanium alloy, with a diameter of 100 μm, a length of 10 mm, and a distance between the two poles of 200 μm. Ultimately, the electrodes were fixed to the skull with dental cement, forming a tight connection with the cranial nail. To verify the accuracy of the electrode implantation sites, after the completion of the rat motor neuromodulation experiments, we performed perfusion and brain tissue sampling on the experimental rats, followed by frozen tissue sectioning and hematoxylin and eosin staining for the histological analysis of the rat brain tissue (Figure 2a,b).

### 2.3. Electrical Stimulation and Behavioral Testing Platform

In this study, the STG4008 electrical stimulator (Multi Channel Systems MCS GmbH, Reutlingen, Germany) was used to construct an electrical stimulation neuromodulation system. The STG4008 is a program-controlled electrical stimulator, which can be easily used to set the stimulation waveforms and waveform parameters through the upper electrical stimulation parameter setting platform, including the stimulation intensity, frequency, number of pulses, and duration. The STG4008 electrical stimulator has a built-in opto-isolator that can output stimulus pulses in either voltage or current mode, making it easy to choose according to the experimental needs. In this study, a constant-current electrical stimulation was used to modulate the locomotion of rats. Additionally, in order to prevent the accumulation of charges caused by single-phase electrical stimulation from damaging the brain tissue, the stimulation waveform was set as a positive–negative symmetric bidirectional wave to balance the total amount of positive and negative charges introduced into the rat brain. Referring to previous studies [11,12], we examined four key parameters relevant to the charge delivered, including the stimulus intensity (amplitude), stimulus frequency, pulse width, and stimulation time (pulse number).

**Figure 3:**
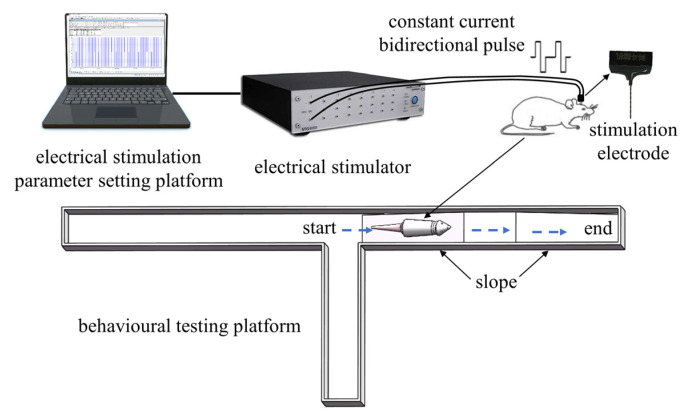
The electrical stimulation and behavioral testing platform. (**top**) The electrical stimulation platform comprises the electrical stimulation parameter setting platform, the STG4008 electrical stimulator, and the stimulation electrodes. (**bottom**) The behavioral testing platform comprises a “T” maze, the ramp, and a Plexon CinePlex motion capture system.

The inclined behavioral testing platform consists of three parts: the main frame, the ramp, and the motion behavior recording system (Figure 3). The main frame and ramp are made of acrylic board, the main frame is a “T” maze, the ramp is placed in the right arm, and the rats are confined to move in the main frame during the experiment. The experimental platform for the turning motion is a circular minefield with a diameter of 1 m. In this study, a dedicated motor behavior recording system was constructed using a Plexon CinePlex motion capture system (Plexon Inc., Dallas, TX, USA), with the camera placed on top of the entire setup to capture the rat’s movement process from top to bottom. The frame rate of the camera was set to 80 Hz during the experiment to ensure that the rat’s movement process could be analyzed offline at a later stage.

### 2.4. Data Analysis

**Figure 4:**
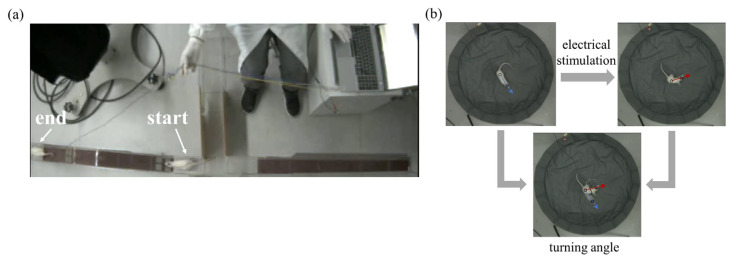
Behavioral tests in rats. (**a**) The climbing behavior test of rats and the locomotion velocity of rats across the inclined platform was used as the evaluation index of the effect about VTA electrical stimulation. (**b**) The turning behavior test of rats and the turning angle were used as the evaluation index of the effect of NS electrical stimulation. The blue arrow represented the direction of the line connecting the center of the trunk and the center of the head before stimulation. The red arrow represented the direction of the line connecting the center of the trunk and the center of the head after stimulation.

In this study, the movement speed of rats on the inclined platform was used as an evaluation index to assess the effect of the electrical stimulation of the VTA of the midbrain in modulating the climbing behavior of rats (Figure 4a). The neuromodulation effect of the rat’s turning movement was evaluated by the turning angle, which was defined as the deviation of the angle from the line connecting the center of the trunk and the center of the head before and after stimulation (Figure 4b). All data are presented as the mean ± standard error of the mean (SEM), and the Wilcoxon rank-sum test was used to evaluate the significant difference. The Wilcoxon rank-sum test is a nonparametric statistical test for testing the difference between unpaired data.

## 3. Results

### 3.1. Inclined Movement Control in Rats Based on Stimulation of the VTA

**Figure 5:**
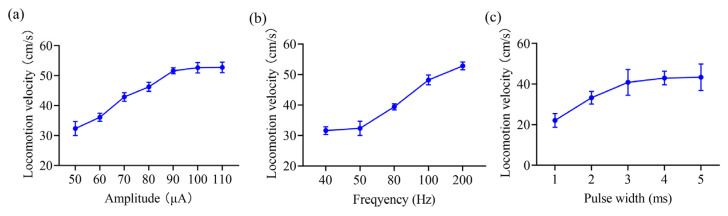
The modulatory effects of inclined movement based on VTA stimulation. (**a**) The effects of varying the stimulation amplitude. (**b**) The effects of varying the stimulation frequency. (**c**) The effects of varying the pulse width.

The results showed that the electrical stimulation of the VTA can induce stable inclined movement in rats. In particular, the inclined movement was strongly synchronized with the electrical stimulation, and the rats immediately stopped the inclined movement when the stimulation ended. We also tested the effect of electrical stimulation intensity on climbing movement in rats, with a stimulation frequency of 50 Hz, a pulse width of 2 ms, and a number of pulses of 250. The results showed that as the stimulus intensity increased, the locomotion velocity of the rats on the slope gradually increased from 32.35 ± 2.20 cm/s at a current amplitude of 50 μA to 51.61 ± 0.98 cm/s at a current amplitude of 90 μA, *p* = 0.032 and Cohen’s d = 0.78 (Figure 5a). Notably, the results also indicated that further increases in current intensity after the rat reached peak locomotion velocity did not result in a significant increase in the modulatory effect.

Similarly to the effect of the stimulus intensity on the modulation of rat locomotion on the slope, an increase in stimulus frequency induced a gradual increase in the locomotion velocity of the rat on the slope. In the stimulus intensity test, the pulse width was set to 2 ms, the current was set to 50 μA, and the number of pulses was set to 250. The results revealed that the locomotion velocity of the rats increased continuously as the stimulation frequency increased from 40 to 200 Hz, from 31.63 ± 1.19 to 52.86 ± 1.23 cm/s, and with *p* = 0.026 and Cohen’s d = 0.81 (Figure 5b).

We then examined the effect of pulse width on the modulation of inclined locomotion in rats by setting the current amplitude to 50 μA, the stimulation frequency to 50 Hz, and the number of pulses to 250. Similarly to the modulatory effect of stimulus intensity on the rats’ inclined movement, the speed of rats on the slope gradually increased with increasing pulse width within the range of 1 to 4 ms, from 22.11 ± 1.36 to 42.99 ± 0.94 cm/s, with *p* = 0.031 and Cohen’s d = 0.76 (Figure 5c). The results also revealed that once the pulse width reached a certain value, further increases in the pulse width did not lead to a significant increase in the locomotion velocity.

**Figure 6:**
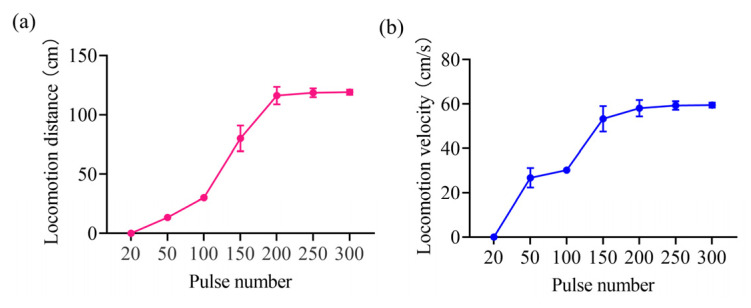
The modulatory effects of varying the pulse number on inclined movement based on VTA stimulation. (**a**) The effects of varying the pulse number on movement distance. (**b**) The effects of varying the pulse number on locomotion velocity.

In addition, we also investigated the effect of the number of stimulation pulses on the modulation of inclined locomotion in rats at a current amplitude of 50 μA, a stimulation frequency of 100 Hz, and a pulse width of 2 ms. The results showed that the number of stimulation pulses could modulate the movement distance of rats on slopes (Figure 6a). When there was no stimulus or the duration of stimulus was too short, rats did not produce inclined movement. When the number of pulses was increased to 20, the head-bobbing exploratory behavior of the rat increased significantly, but there was no limb movement. When the number of pulses was increased to 50, the rats moved small distances; when the number of pulses was increased to 100, the distance the rats traveled on the slope increased. Finally, when the number of pulses was increased to 200 or above, the rats were able to move quickly through the entire slope. The results also showed that the number of pulses had no significant effect on the movement speed of the rats, with significant differences only between small distances and the entire slope (Figure 6b).

### 3.2. Turning Movement Control in Rats Based on NS Pathway Stimulation

**Figure 7:**
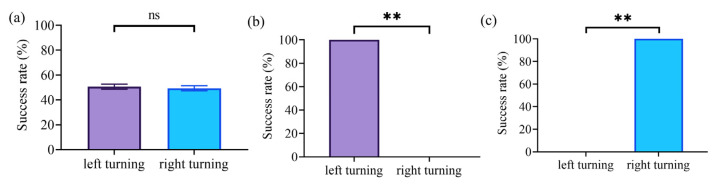
Turning movement control in rats based on the electrical stimulation of the NS pathway. (**a**) Turning success rate of rats without stimulation of the NS pathway. (**b**) Turning success rate of rats with stimulation of the right NS region. (**c**) Turning success rate of rats with stimulation of the left NS region. (** indicates *p* ≤ 0.01, ns indicates no significant difference).

We first tested the effect of the electrical stimulation of the NS pathway to control steering movements in rats. For the test, the stimulation frequency was set to 50 Hz, the current amplitude to 70 μA, the pulse width to 2 ms, and the number of pulses to 200. The results showed that the electrical stimulation of the NS pathway could induce immediate turning movements in rats. Specifically, the electrical stimulation of the right NS region could induce left-turning movement (Figure 7b), and the electrical stimulation of the left NS region could induce right-turning movement (Figure 7c). However, there was no significant difference in the success rate of left/right turning in the absence of electrical stimulation (Figure 7a).

**Figure 8:**
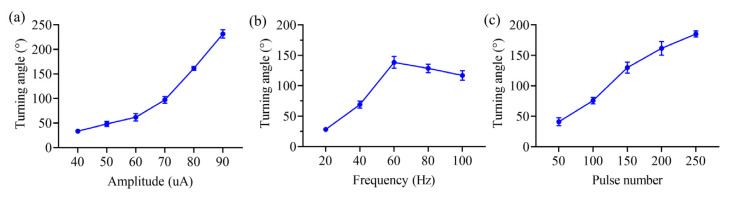
The modulatory effects on the turning locomotor movements based on the electrical stimulation of the NS pathway. (**a**) The effects of varying the stimulation amplitude. (**b**) The effects of varying the stimulation frequency. (**c**) The effects of varying the number of pulses.

We also tested the effect of the electrical stimulation intensity on the turning of locomotor movements in rats, with the stimulation frequency set at 50 Hz, pulse width at 2 ms, and number of pulses at 200. The results showed that as the current amplitude increased, the rats’ turning angle gradually increased from 33.5 ± 4.03°, at a current amplitude of 40 μA, to 231.5 ± 7.65° at a current amplitude of 90 μA, *p* = 0.016, and Cohen’s d = 0.89, with the maximum steering angle exceeding even 180° (Figure 8a).

Unexpectedly, the effect of the stimulus frequency on the turning of locomotor movements showed a different trend compared to stimulus intensity. These results demonstrated that the turning angle of the rats gradually increased with increasing stimulation frequency in the range of 20 to 60 Hz from 28.17 ± 3.24° to 138.44 ± 9.12°, with *p* = 0.013 and Cohen’s d = 0.85 (Figure 8b). At a frequency higher than 60 Hz, the turning angle of the rats slowly decreased as the frequency increased, indicating an inhibitory effect on the turning of locomotor movement, decreasing from 138.44 ± 9.12°, at a stimulation frequency of 60 Hz, to 116.85 ± 7.73° at a stimulation frequency of 100 Hz, with *p* = 0.047 and Cohen’s d = 0.76 (Figure 8b).

Similarly to the effect of the stimulus intensity on the modulation of the turning locomotor movement in rats, the turning angle of the rats gradually increased with an increasing number of stimulation pulses, increasing from 41.13 ± 6.42° at a pulse number of 50 to 185 ± 5° at a pulse number of 250, with *p* = 0.016 and Cohen’s d = 0.82 (Figure 8c).

## 4. Discussion

Previous studies have shown that a successful rat robot system can be created by electrically stimulating the MFB as a reward and electrically stimulating the S1BF as a virtual directional cue for steering [7]. The stimulation of the S1BF mimicked somatosensory inputs to the head and face (especially the whisker area) of rats that tended to turn when they encountered boundaries or obstacles [28]. However, the control of this rat robot turning behavior is highly dependent on the training process. The rat must adapt to the electrical stimulation of the barrel cortex and learn to associate this stimulus with the correct turning direction. This type of turning behavior training relies heavily on the extensive experience of the instructor and is more time-consuming. Trainers must choose reward conditions very carefully to avoid inadvertently rewarding inappropriate behaviors. It takes 1–2 weeks for rats to adapt to steering commands. The steering behavior of the rat robots also fades slowly. Therefore, retraining every few days is necessary to increase the training effect. While a well-trained BF rat will stop navigating for more than a week, it will need to re-adapt to electrical stimulation before navigating well again in a complicated environment. Therefore, the training process severely limited the use and development of this bio-robot. In addition, it is difficult to precisely control the steering angle because of the reliance on the virtual tactile sensation of the rat. This means that there is no strong correlation between electrical stimulation parameters and steering angle.

Another method of controlling steering behavior is to electrically stimulate the ventral posteromedial (VPM) to generate virtual touch sensation [12]. This sense of touch is a more “solid” directional cue for rats than the directional cue from whiskers. The electrical stimulation of the VPM could induce immediate turning movements in rats. This method eliminates the training process and greatly saves time and labor. The VPM stimulation method also demonstrates the potential to quantitatively control the turning angle by adjusting different combinations of electrical stimulation parameters. However, there are also some limitations, such as continuous electrical stimulation inhibiting steering behavior. In addition, different rats may exhibit different steering directions [11].

In order to address these problems, we developed a new method to gain control of inclined locomotor movements in rats by the electrical stimulation of the VTA of the midbrain and motor control of steering in rats by the electrical stimulation of the NS pathway. On this basis, the effects of electrical stimulation parameters on the control of motor function were quantitatively evaluated. The results showed that the electrical stimulation of the rat VTA could induce stable inclined movement in rats and that the neuromodulatory effect significantly correlated with the electrical stimulation parameters. In addition, the electrical stimulation of the NS pathway was able to directly and stably induce the steering movements of the head and trunk to the contralateral side of the stimulated side of the rat. This method also requires no training and greatly improves the efficiency of building rat robots. In addition, our results also demonstrate the potential to quantitatively control the locomotion velocity and steering angle by modulating electrical stimulation parameters. The comparative analysis of three rat robot construction methods is shown in Table 1.

### 4.1. Electrical Stimulation of the VTA Induces Stable Inclined Movement

Our results showed that the electrical stimulation of the VTA can induce stable inclined movements in rats, suggesting that the electrical stimulation of the VTA can produce similar effects to the electrical stimulation of the MFB. Research on rat robotics has shown that the electrical stimulation of the MFB induces forward movements in rats [7]. Our study further demonstrated that the electrical stimulation of the VTA can induce unconventional uphill and downhill walking in rats. Previous studies have confirmed that VTA DA neurons play a direct regulatory role in movement and reinforcement [29], which project to the large area of the primary motor cortex [30,31]. Further studies have shown that the VTA projects indirectly to the spinal cord and that the activation of the VTA modulates the activity in the ipsilateral motor cortex, generating muscle responses in the contralateral forelimb [32]. In addition, VTA DA and non-DA neurons conjunctively process locomotor-related motivational signals that are associated with movement initiation, maintenance, and termination [33]. Thus, the induction of inclined movements by VTA stimulation may be due to a combination of motor control and motivational reinforcement.

Our study also found that as the stimulus intensity increased, the locomotion velocity of the rats on the slope gradually increased (Figure 5a). Additionally, this study revealed that the increase in current intensity essentially increases the total charge injected into the VTA. Thus, one possible explanation is that the increase in the charge activated more VTA DA neurons, leading to an increase in DA release, which in turn induced a stronger motor intention in the rats. In addition, it has been shown that more movement-sensitive gamma-aminobutyric acid (GABA)ergic neurons in the VTA are activated [34]. Unexpectedly, our results also showed that once the rat’s movement speed had peaked, further increases in current intensity did not lead to a significant increase in the modulatory effect (Figure 5a). This may be due to the saturation of the neurons activated at the current amplitude of 90 μA; thus, further increases in current intensity do not activate more VTA neurons. Overall, the modulation of the inclined locomotor function in rats can be achieved by changes in stimulus intensity.

A reported study has indicated that VTA neurons could increase their firing rates as the velocity of rats increased, encoding both the acquisition of reward and the movements made to obtain it [35]. This is consistent with our findings which indicated that as the stimulus frequency increased, the locomotion velocity of the rats on the slope gradually increased (Figure 5b). It is possible that the increase in stimulus frequency induced an increase in the firing rates of VTA neurons, which encode both the details of the movement and the motivation for the behaviors. Overall, the modulation of the inclined locomotor function in rats can be achieved by changes in stimulation frequency.

Similarly to increasing current amplitude, increasing pulse width essentially increases the total charge injected into the VTA, thereby activating a greater number of VTA neurons (Figure 5c). Notably, the results also showed that once the pulse width reached a certain value, further increases in pulse width did not result in a significant increase in movement velocity. The reason for this result may be that the VTA neurons activated were saturated at a pulse width of 4 ms, and a continued increase in charge does not continue to activate more neurons. Overall, the modulation of the inclined locomotor function in rats can be achieved by changes in the stimulation pulse width.

Our results also showed that the number of stimulation pulses could modulate the movement distance of rats on slopes (Figure 6a). The primary effect of increasing the number of pulses was to increase the duration of the electrical stimulation; thus, it can be concluded that the stimulus duration modulates the rat’s movement distance on the slope. The results also revealed that the number of pulses had no significant effect on the movement velocity, with significant differences only between small distances and the entire slope (Figure 6b). The main reason for this may be that when the rats were moving during short distances, they were still in the exploration stage of locomotor excitation, and the activity of VTA neurons mainly regulates exploratory behavior, whereas when the number of pulses is sufficient, a large number of VTA neurons are stimulated to control the locomotor behavior. Overall, the movement distance of rats on slopes can be modulated by varying the number of pulses.

The results of the histological staining analysis of rat brain tissues showed that the electrodes were correctly implanted in the VTA (Figure 2a), consistent with the previously identified electrode implantation sites, further confirming that the motor control of climbing in rats in the present study was induced by modulating the electrical stimulation of the VTA.

### 4.2. Electrical Stimulation of the NS Pathway Induces Immediate Turning Movements

The NS pathway is a DA pathway in the brain that plays a key role in regulating motor and reward functions as part of the basal ganglia circuit [21,22,23,24]. A previous study has shown that the unilateral activation of the dopaminergic NS pathway causes rodents to turn away from the activated side, thus elucidating the functional role of the NS pathway in controlling turning movements [36,37]. Further research has demonstrated that the continuous electrical stimulation of ascending axons in the posterolateral hypothalamus close to the NS dopaminergic neurons induced the rats to rapidly circle to the unstimulated side, and the circling behavior was inhibited by drugs that deplete brain DA as well as drugs that block DA receptors [26]. In addition, in experiments with cats, the electrical stimulation of the substantia nigra also produced head-turning movements followed by reverse circling [27]. Our study further demonstrated that in rats, the electrical stimulation of the NS pathway can directly and consistently induce head and trunk steering movements to the contralateral side of the stimulus, and thus, the steering control was achieved in all rats in the experiment. (Figure 7b,c).

Our results also revealed that as the current amplitude increased, the rat turning angle gradually increased (Figure 8a). Keeping other stimulus parameters constant, the increase in current intensity essentially increases the total charge applied to the NS pathway. Since the NS pathway consists mainly of nerve fibers between the substantia nigra and the neostriatum, it can be inferred that the increase in charge likely activated more striatal neurons, which play an important role in motor control, thus inducing steering movements in the rats. At the same time, more NS neurons were activated, leading to increased DA release. Overall, the modulation of rat steering locomotor function can be achieved by varying the stimulus current amplitude.

Additionally, the results also demonstrated that the turning angle of the rats gradually increased with increasing stimulation frequency in the range of 20 to 60 Hz (Figure 8b). However, when the frequency was higher than 60 Hz, the turning angle of the rats decreased slowly as the frequency increased, indicating an inhibitory effect on the turning movement. The enhanced effect of electrical stimulation frequency on the modulation of steering movements may be due to an increase in the response frequency of striatal neurons with increasing stimulation frequency, leading to an increase in the number of activated neurons per unit time and an enhancement of the motor control effect. The inhibitory effect may be due to the reduced substantia nigra response to high-frequency electrical stimulation, the relative decrease in DA release, and the relative decrease in the rat’s intention to control movement.

Like the effect of stimulus intensity on the modulation of the rat turning movement, the turning angle of the rats gradually increased with the increasing number of stimulation pulses (Figure 8c). The main effect of increasing the number of pulses was to increase the duration of electrical stimulation, thereby inducing a sustained firing of striatal neurons. Overall, the modulation of the rat steering locomotor function can also be achieved by varying the number of stimulus pulses.

The results of the histological staining analysis of rat brain tissues showed that the electrodes were correctly implanted within the NS pathway of the rat substantia nigra striata bundle (Figure 2b), which is consistent with the previously identified electrode implantation site, further confirming that the control of the rats’ steering movements in this study was induced by the electrical stimulation of the NS pathway modulation.

## 5. Conclusions

In this study, we developed a new method to gain control of inclined movement in rats by the electrical stimulation of the VTA, as well as motor control of turning in rats by the electrical stimulation of the NS pathway. The results showed that the electrical stimulation of the VTA can induce stable inclined movements in rats and that the neuromodulatory effect was significantly correlated with the electrical stimulation parameters. Additionally, the electrical stimulation of the NS pathway could directly and stably induce the steering movements of the head and trunk of the rat to the contralateral side of the stimulated side, and steering control was achieved in all rats in the experiment. These findings are of great importance for the motor control of brain-controlled rats, especially in a field environment with many slopes. Moreover, the rat robot constructed based on this new method does not require pre-training while ensuring reliability, which greatly improves the preparation efficiency and has certain practical application value.

Compared with traditional robots, rat robots possess notable advantages in terms of environmental adaptability, mobility flexibility, and energy supply. However, ethical limitations must also be considered. The construction of rat robots must adhere to strict ethical restrictions to guarantee the welfare of animals. Furthermore, when applying rat robots in real-world environments, the reliability is a critical consideration. Prolonged electrical stimulation may result in stimulus adaptation, which can lead to the inability of the electrical stimulation to elicit the target behavior. Therefore, the choice of electrical stimulation parameters is particularly important to ensure effective control while avoiding prolonged high-intensity electrical stimulation. In terms of the long-term sustainability of behaviors, it is particularly important to consider the issue of reduced electrode efficacy due to immune reactions. The development of new material electrodes that do not elicit an immune response may be an effective means of addressing this issue.

Although control of the rat’s turning and climbing movements was achieved, it still required the manual real-time transmission of control commands, which was less efficient. In addition, the electrical stimuli were delivered via wires, which restricted the rats’ freedom of movement to some extent. Future research will focus on (1) the wireless delivery of electrical stimuli and (2) the construction of a closed-loop system by installing sensors to detect the environmental state and the locomotor behavior of the rat and to select the parameters of the electrical stimulation automatically to gain automated control of the locomotor behavior of the rat.

## Figures and Tables

**Table 1:** The comparative analysis of three rat robot construction methods.

	Training Procedure	Accuracy	Steering Consistency	Long-Term Stability
S1BF [7]	1–2 weeks	No strong correlation between steering angle and stimulus parameters	More consistent steering direction	about one week
VPM [11,12]	no training	Steering angle is parametrically controllable, but continuous electrical stimulation inhibits steering behavior in rats.	The VPM stimulation may create contralateral or ipsilateral turning behaviors.	about one month
Our method	no training	Steering angle is parametrically controllable.	More consistent steering direction	about one month

## Data Availability

The data and materials supporting the results in this article are available from the corresponding author on reasonable request due to privacy and ethical restrictions.
